# The Molecular Mechanisms of Actions, Effects, and Clinical Implications of PARP Inhibitors in Epithelial Ovarian Cancers: A Systematic Review

**DOI:** 10.3390/ijms23158125

**Published:** 2022-07-23

**Authors:** Chien-Hui Lau, Kok-Min Seow, Kuo-Hu Chen

**Affiliations:** 1 Department of Obstetrics and Gynecology, Taipei Tzu-Chi Hospital, The Buddhist Tzu-Chi Medical Foundation, Taipei 231, Taiwan; qianhuilau0725@gmail.com; 2 Department of Obstetrics and Gynecology, Shin Kong Wu Ho-Su Memorial Hospital, Taipei 111, Taiwan; m002249@ms.skh.org.tw; 3 Department of Obstetrics and Gynecology, National Yang-Ming Chiao-Tung University, Taipei 112, Taiwan; 4 School of Medicine, Tzu-Chi University, Hualien 970, Taiwan

**Keywords:** ovarian cancer, PARP inhibitor, olaparib, rucaparib, niraparib

## Abstract

Ovarian cancer is the most lethal gynecologic malignancy in the United States. Some patients affected by ovarian cancers often present genome instability with one or more of the defects in DNA repair pathways, particularly in homologous recombination (HR), which is strictly linked to mutations in breast cancer susceptibility gene 1 (BRCA 1) or breast cancer susceptibility gene 2 (BRCA 2). The treatment of ovarian cancer remains a challenge, and the majority of patients with advanced-stage ovarian cancers experience relapse and require additional treatment despite initial therapy, including optimal cytoreductive surgery (CRS) and platinum-based chemotherapy. Targeted therapy at DNA repair genes has become a unique strategy to combat homologous recombination-deficient (HRD) cancers in recent years. Poly (ADP-ribose) polymerase (PARP), a family of proteins, plays an important role in DNA damage repair, genome stability, and apoptosis of cancer cells, especially in HRD cancers. PARP inhibitors (PARPi) have been reported to be highly effective and low-toxicity drugs that will tremendously benefit patients with HRD (i.e., BRCA 1/2 mutated) epithelial ovarian cancer (EOC) by blocking the DNA repair pathways and inducing apoptosis of cancer cells. PARP inhibitors compete with NAD^+^ at the catalytic domain (CAT) of PARP to block PARP catalytic activity and the formation of PAR polymers. These effects compromise the cellular ability to overcome DNA SSB damage. The process of HR, an essential error-free pathway to repair DNA DSBs during cell replication, will be blocked in the condition of BRCA 1/2 mutations. The PARP-associated HR pathway can also be partially interrupted by using PARP inhibitors. Grossly, PARP inhibitors have demonstrated some therapeutic benefits in many randomized phase II and III trials when combined with the standard CRS for advanced EOCs. However, similar to other chemotherapy agents, PARP inhibitors have different clinical indications and toxicity profiles and also face drug resistance, which has become a major challenge. In high-grade epithelial ovarian cancers, the cancer cells under hypoxia- or drug-induced stress have the capacity to become polyploidy giant cancer cells (PGCCs), which can survive the attack of chemotherapeutic agents and start endoreplication. These stem-like, self-renewing PGCCs generate mutations to alter the expression/function of kinases, p53, and stem cell markers, and diploid daughter cells can exhibit drug resistance and facilitate tumor growth and metastasis. In this review, we discuss the underlying molecular mechanisms of PARP inhibitors and the results from the clinical studies that investigated the effects of the FDA-approved PARP inhibitors olaparib, rucaparib, and niraparib. We also review the current research progress on PARP inhibitors, their safety, and their combined usage with antiangiogenic agents. Nevertheless, many unknown aspects of PARP inhibitors, including detailed mechanisms of actions, along with the effectiveness and safety of the treatment of EOCs, warrant further investigation.

## 1. Introduction

Ovarian cancer is the second most common gynecologic malignancy and the most common cause of gynecologic cancer death in the United States [1]. The treatment and prognosis of ovarian cancer depend on the specific tumor histopathologic type. Among all types of ovarian cancers, epithelial ovarian cancers (EOC)s comprise the majority (about 95%); the remainders are malignant germ cell and sex cord-stromal cell tumors. The subtypes of EOCs include high-grade serous, low-grade serous, endometrioid, clear cell, and mucinous ovarian cancers, in which serous cancer is the most common subtype (75%) [2]. The majority of patients with advanced-stage ovarian cancers experience relapse and require additional treatment after initial therapy, including an optimal cytoreductive debulking surgery and platinum-based chemotherapy [3]. Because traditional chemotherapies have therapeutic limitations and cumulative toxicities in the treatment of ovarian cancers, other types of therapies are considered second-line treatments for maintenance or prevention. In general, genome instability is a hallmark of ovarian cancer. Some patients affected by ovarian cancers often present genome instability with one or more of the defects in DNA repair pathways, particularly in homologous recombination (HR), which is strictly linked to mutations in breast cancer susceptibility gene 1 (BRCA 1) or breast cancer susceptibility gene 2 (BRCA 2) [4,5]. An estimated 20% of EOC patients are associated with mutations in BRCA 1 or BRCA 2, which relate to homologous recombination deficiency (HRD). Up to 50% of high-grade serous ovarian cancers (HGSOC) present with HRD [6].

DNA damages are frequent during cell life cycles, which result from single-strand break (SSB) or double-strand break (DSB) that can cause genome instability and cell death if not correctly repaired. There are at least five major DNA repair pathways in humans: base excision repair (BER), nucleotide excision repair (NER), mismatch repair (MMR), homologous recombination (HR), and non-homologous end joining (NHEJ), which are active throughout different stages of the cell cycle, allowing the cells to repair the DNA damages. Base excision repair (BER) is responsible for repairing SSBs, while homologous recombination (HR) and non-homologous end joining (NHEJ) are two main pathways in repairing DSBs. The absence, reduction, or dysfunction of proteins involved in these pathways may lead to cellular implications, determining mutagenesis, toxicity, and cell death [7,8].

Poly (ADP-ribose) polymerase (PARP) plays a dominant role in DNA single-strand break repair (SSBR). Inhibition of PARPs may cause both SSBR deficiency and HRD in BRCA 1/2-deficient patients, leading to cell death. Based on the molecular mechanisms of actions that target the key points of cancer cells in DNA repair pathways, PARP inhibitors (PARPi) emerge as a new therapeutic approach in the management of EOCs, particularly for HRD (i.e., BRCA 1/2 mutated) ovarian cancers through the mechanism of synthetic lethality [9]. By blocking the DNA repair pathways and inducing apoptosis of cancer cells, PARPi has been reported to be a highly effective and low-toxicity drug that will tremendously benefit patients with HRD ovarian cancers [10]. Currently, three PARP inhibitors olaparib, niraparib, and rucaparib have been approved by the Food and Drug Administration (FDA) for clinical use in EOC patients, though with different clinical indications and toxicity profiles. However, similar to other chemotherapy agents, PARP inhibitors also face drug resistance, which has become a major challenge [11,12].

In this review, we discuss the underlying molecular mechanisms of PARP and PARP inhibitors and the results from the clinical studies that investigated the effects of the FDA-approved PARP inhibitors on epithelial ovarian cancers. We also summarize the current research progress on PARP inhibitors, their safety, and their combined usage with antiangiogenic agents.

## 2. Methods

The literature was searched for basic and clinical studies that investigated the usage of PARP inhibitors in epithelial ovarian cancers. All articles were solicited from the databases Medline and PubMed using the searching terms “ovarian cancer”, “PARP inhibitor”, “olaparib”, “rucaparib”, and “niraparib” for the topic. For screening and selection in the next stage, only full-text articles were considered for inclusion in further analyses. To ensure the novelty of this review, the articles published prior to 2005 were excluded. From a total of 93 articles identified in the searching process, 40 articles met the criteria for inclusion.

**Figure 1:**
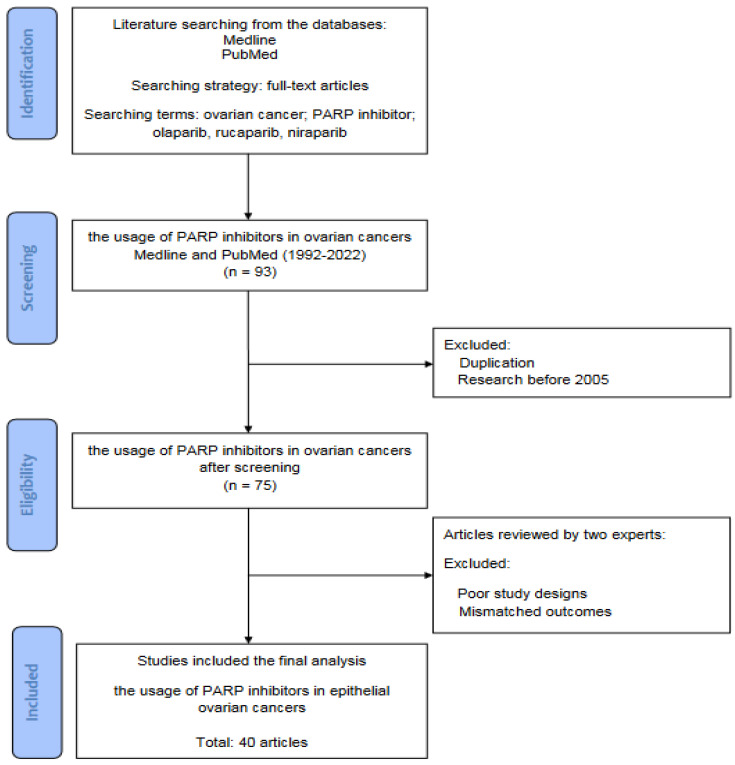
The flowchart of database searching, screening, selection, and inclusion of eligible articles from the literature.

Figure 1 is a flowchart of the searching, screening, and including process of the references we selected from the literature. In this review, all of the reference articles were retrieved from the databases Medline and PubMed using the searching terms “ovarian cancer”, “PARP inhibitor”, “olaparib”, “rucaparib”, and “niraparib” for the research topic. In this stage, only full-text articles were considered to be included for further analysis. Up to 31 March 2022, we searched potential articles in the literature from the databases Medline and PubMed. In the second stage, duplicated articles and articles prior to 2005 were excluded after screening. The literature published from 1 January 1992 to 31 March 2022 has been searched to identify eligible articles for review. In the next stage, two experts in the field independently inspected the contents of articles, including demographics, research designs, and outcomes, and identified potentially eligible studies for inclusion. The retrieved articles with poor research designs or mismatched outcomes were excluded at this stage. The discrepancies between the two experts were discussed through their mutual communication to reach a consensus. All eligible articles were included in the review using the searching terms and strategies (database searching, screening, selection, and inclusion of eligible articles). Ultimately, a total of 40 articles were collected for review.

## 3. Molecular Mechanisms of PARP and PARPi Actions

### 3.1. The PARP Enzymes

**Figure 2:**
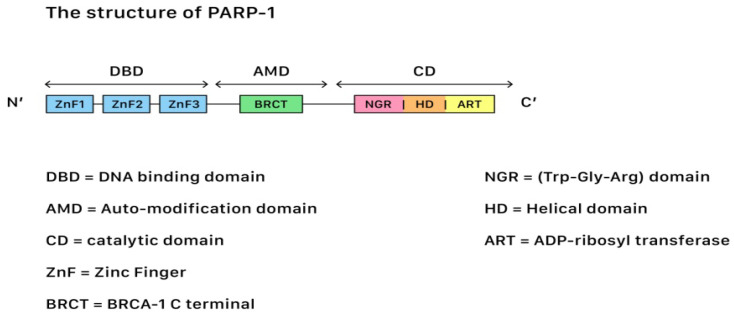
The structure of PARP-1, showing the segments and corresponding functions.

In terms of genetic repair for DNA damages, Poly (ADP-ribose) polymerases (PARP) belong to a family of 17 proteins that play critical roles in different stages of cellular life cycles, including the stress response, chromatin modification, DNA repair, and apoptosis induction (cell death). Among all kinds of PARP, PARP-1 is the most important member due to its role in the DNA repair pathways. Figure 2 shows the structure of PARP-1.

PARP-1 contains three functional domains: a N-terminus DNA-binding domain with zinc finger motifs, a central auto-modification domain that is responsible for releasing the protein from the DNA after catalysis, and a C-terminus catalytic domain (CAT) that houses the protein enzymatic activity and substrate binding sites for nicotinamide-adenine-dinucleotide (NAD^+^). SSBs often occur after oxidative damages, while DSBs often occur during damages that originate from the injuries of chemical or physical agents. The repair of both SSBs and DSBs is closely related to the PARP actions.

### 3.2. The Mechanism Underlying Single-Strand Break Repair (SSBR) of DNAs

**Figure 3:**
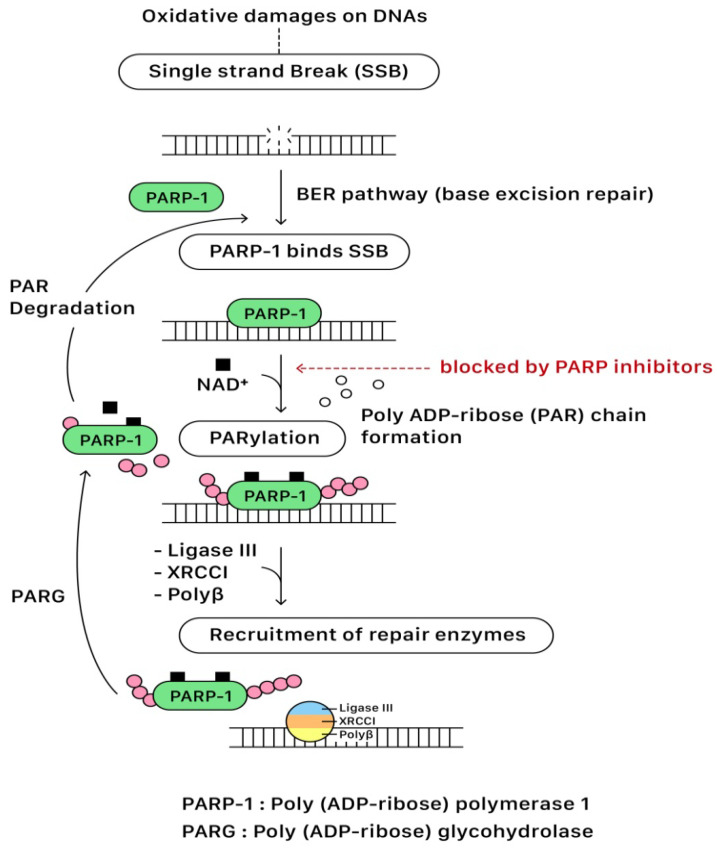
The base excision repair (BER) pathway of PARP for repairing SSB.

In the presence of DNA single-strand break (SSB), the DNA-binding domain of PARP binds to the DNA SSB site. Following the activation of NAD^+^ on the catalytic domain (CAT), PARP initiates the synthesis of polymeric adenosine diphosphate-ribose (Poly (ADP-ribose) or PAR) chain that plays an essential role in recruiting the repairing enzymes for the DNA repair pathway. The targeted enzymes include DNA ligase III, X-ray cross-complementing gene 1 (XRCC1), and DNA polymerase beta (polβ). After repairing SSB, the PAR chains are degraded via Poly (ADP-ribose) glycohydrolase (PARG). Eventually, PARP and repairing complexes dissociate from the DNA for recycling in the future [13,14]. The base excision repair (BER) pathway of PARP for repairing SBB is illustrated in Figure 3.

### 3.3. The Mechanism Underlying Double-Strand Break Repair (DSBR) of DNAs

On the other hand, PARP can also modulate the pathways of DNA double-strand break repair (DSBR) through the recruitment of DSB repair enzymes MRE11 and NBS1, which play key roles in homologous recombination (HR). In addition, PARP also regulates the expression of important HR genes BRCA 1 and RAD51 at a transcription level.

**Figure 4:**
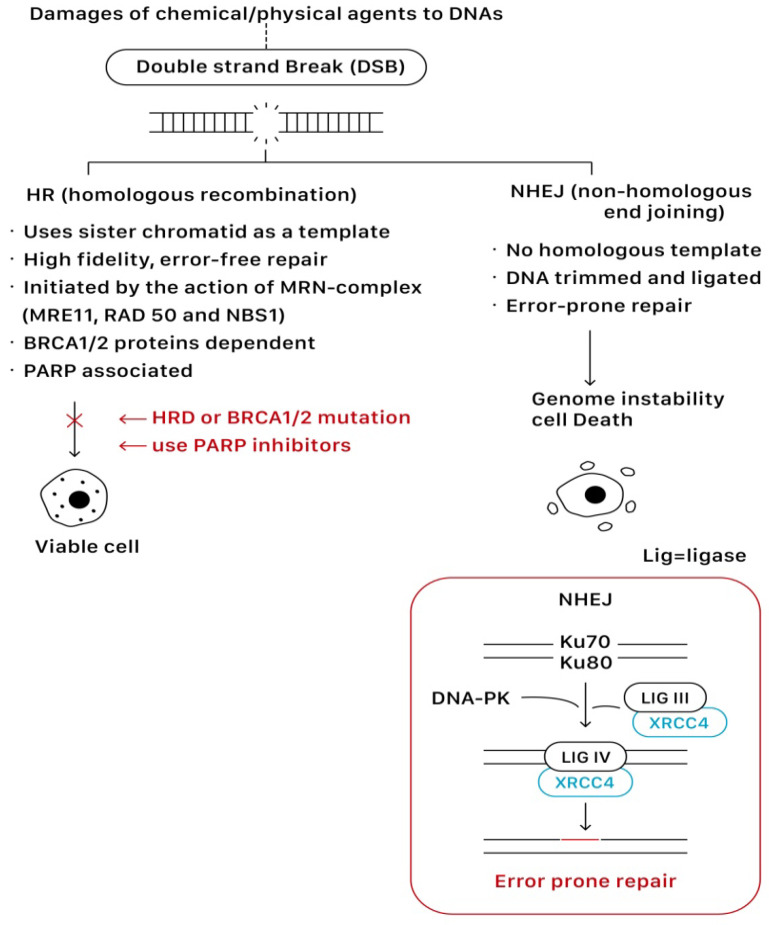
The repairing process of DSBs via the HR and NHEJ pathways.

Homologous recombination (HR) and non-homologous end joining (NHEJ) are two main pathways to resolve double-strand breaks (DSBs). HR predominates instead as a mechanism of DNA repair during the S or G2 phase of the cell cycle when a sister chromatid is available to use as a template for in situ repair, in which nucleotide sequences are exchanged between two similar or identical molecules of DNA. It is a high-fidelity, error-free form of DNA repair mechanism and requires functional BRCA proteins for repairing DSBs. In contrast, NHEJ is active throughout all phases of the cell cycle, and it does not require a homologous template that the DNA is simply trimmed and ligated. Given the lack of a contrast template strand, NHEJ is a faster but error-prone mechanism of DNA repair [9,15]. This error-prone DNA repair often predisposes to genome instability and cell death. Figure 4 shows the repairing process of DSBs via the HR and NHEJ pathways.

### 3.4. BRCA 1 and BRCA 2

BRCA 1 and BRCA 2 are critical proteins involved in the HR of DSBR [16].

The MRN complex, composed of MRE11, RAD50, and NBS1, plays an important role in response to DNA damage, while the most well-recognized is as a sensor of DSB to initiate HR. The MRN complex recognizes and binds the DSB to facilitate the recruitment and activation of ATM and ATR kinases that phosphorylate downstream targets, including CHEK2, P53, BRCA1, and H2AX. BRCA 1, assisted by BARD1 and BRIP1, has an active role in signaling DNA damage and in cell-cycle checkpoint regulation that organizes the remaining proteins to the site of repair. The MRN complex then initiates the 5′ to 3′ nucleolytic resection of the DNA to form 3′ overhangs of single-stranded DNA that are bound by RPA. With the assistance of PALB2, BRCA 2 recruits and loads an essential recombination enzyme, RAD51, onto RPA-coated DNA. The RAD51 then invades the homologous DNA strand in a process called strand invasion, allowing the remaining DNA repair to occur with the use of the sister chromatid as the template for error-free repair [9].

### 3.5. PARP Inhibitors: The Mechanisms of Actions

PARP inhibitors compete with NAD^+^ at the catalytic domain (CAT) of PARP to block PARP catalytic activity and the formation of PAR polymers. These effects compromise the cellular ability to overcome DNA SSB damage (Figure 3). On the other hand, the unrepaired SSBs can be converted to DSBs through the collapse of the replication fork when PARP is inhibited (PARP trapping mechanism). As previously discussed, homologous recombination (HR) is the essential error-free pathway to repair the DNA of DSBs during cell replication, and it requires functional BRCA 1/2 proteins. The process of HR will be blocked in the condition of BRCA 1/2 mutations. The PARP-associated HR pathway can also be partially interrupted by using PARP inhibitors (Figure 4).

The absence of either a functioning base excision repair (BER) pathway or homologous recombination (i.e., HR deficiency or BRCA 1/2 mutations) has no detrimental impact on cell viability, but the deficiency of both together can lead to synthetically lethal death. Untreated cancer cells without BRCA mutation (HR+, BER+), cancer cells without BRCA mutation treated with a PARP inhibitor (HR+, BER−), and untreated BRCA-deficient cancer cells (HR−, BER+) are all viable conditions. However, homologous recombination-deficient (HRD) cancer cells treated with a PARP inhibitor (HR−, BER−) are selectively targeted for synthetically lethal cell death as the DNA repair of both SSBs and DSBs relies above all on NHEJ, the error-prone repair pathway [16]. PARP inhibitors have been developed for targeting cancers related to BRCA 1/2 gene mutations. Table 1 depicts the presence or deficiency of HR and BER on the outcomes of non-mutated or BRCA 1/2 mutated cancer cells treated with/without PARP inhibitors.

## 4. Therapeutic Effects of PARP Inhibitors in Ovarian Cancers: Clinical Trials

Poly (ADP-ribose) polymerase inhibitors (PARPi) block the repair of DNA SSBs. For ovarian cancers associated with BRCA mutations or HRD, they result in cell death due to inefficiencies in DNA cell repair mechanisms. PARP inhibitors have been widely studied in the past decade, and the results are highly promising. To date, three PARP inhibitors, namely olaparib, rucaparib, and niraparib, have been approved by the FDA for multiple indications in ovarian cancers. The approval history, indications, clinical usage, and associated medical studies are discussed below. Table 2 is a summary of randomized trials of PAPR inhibitors, including olaparib, rucaparib, and niraparib.

### 4.1. Olaparib

Olaparib is the first PARP inhibitor introduced in clinical practice and has been used for both the maintenance and treatment of ovarian cancers based on a few highly successful clinical trial studies. It was first approved by the FDA in December 2014 as a monotherapy for the treatment of ovarian cancers in germline BRCA-mutated (gBRCAm) patients who have undergone at least three prior lines of chemotherapy, according to the results of a large single-arm, phase II study—Study 42. In this study, an oral dose of olaparib 400 mg BID was given as a monotherapy in 298 patients with gBRCAm ovarian, breast, prostate, and pancreatic cancers who had received at least three prior lines of chemotherapy, of whom 193 patients had recurrent ovarian cancers. In patients with gBRCA1/2m recurrent ovarian cancers, 137/193 had measurable diseases at baseline. Eligible patients were treated continuously until disease progression or other olaparib discontinuation criteria were met. After olaparib treatment, the objective response rate (ORR) was 34% (46/137; 95% confidence interval (CI): 26–42%) and the median duration of response (DOR) was 7.9 months (95% CI: 5.6 months–9.6 months) [17,18].

In 2017, the olaparib monotherapy was approved by the FDA for maintenance treatment for platinum-sensitive, relapsed ovarian cancers regardless of the presence or absence of BRCA mutations, based on the results of two international, randomized, double-blind, placebo-controlled clinical trials known as Study 19 and SOLO-2, at the dose of 400 mg BID and 300 mg BID, respectively. All patients in both trials had been pre-treated with at least two prior platinum-based chemotherapies. Study 19 was a phase II trial using olaparib as maintenance therapy in relapsed high-grade serous ovarian cancers (HGSOC), fallopian tubes, or primary peritoneal cancers. Notably, the BRCA status (with/without BRAC mutation) was not applied for patient enrollment in this study. A total of 265 patients were randomly assigned to receive either olaparib (*n* = 136) at a dose of 400 mg BID or placebo (*n* = 129) after a partial or complete response to their most recent platinum-based chemotherapy. The median progression-free survival (PFS) was significantly longer in the olaparib group than in the placebo group (8.4 months vs. 4.8 months; hazard ratio (HR): 0.35; 95% CI: 0.25–0.49; *p* < 0.001) [19].

SOLO-2 is a phase III trial to evaluate the effect of maintenance treatment with olaparib in BRCA 1/2 mutated patients with platinum-sensitive relapsed ovarian, fallopian tube, and primitive peritoneal cancers. A total of 295 patients were randomized to receive either olaparib (*n* = 196) at a dose of 300 mg BID or placebo (*n* = 99) after a partial or complete response to their most recent platinum-chemotherapy. The patients with gBRCAm showed significant improvement in the median PFS when treated with olaparib tablets compared with those treated with the placebo (19.1 months vs. 5.5 months; HR: 0.30; 95% CI: 0.22–0.41, *p* < 0.0001) [20]. In the subsequent report, the median overall survival (OS) was 52 months (95% CI: 41.5 months–59.1 months) in the olaparib group and 39 months (95% CI: 31.4 months–48.6 months) in the placebo group (HR: 0.74, 95% CI: 0.54–1.00) at a median follow-up of 65 months [21].

Based on the randomized, double-blind, phase III SOLO-1 trial, the monotherapy of oparalib has furthermore obtained approval to be used for maintenance treatment in patients with newly diagnosed advanced (FIGO stage III–IV), high-grade, *BRCA*-associated EOCs who have a partial or complete clinical response to first-line platinum-based chemotherapy. In this trial, 391 patients were randomly assigned to either maintenance with olaparib (*n* = 260) at a dose of 300 mg BID or placebo (*n* = 131); the patient in both groups had been diagnosed with advanced (FIGO stage III–IV), high-grade, *BRCA*-associated serous or endometrioid ovarian, fallopian tube or primary peritoneal cancers who had a partial or complete clinical response to platinum-based chemotherapy. A total of 388 patients had a centrally confirmed gBRCAm, and no patients received bevacizumab. After a median follow-up of 41 months, the monotherapy of olaparib resulted in a lower three-year rate of disease progression or death compared with the placebo therapy (60% vs. 27%; HR: 0.30, 95% CI: 0.23–0.41, *p* < 0.0001), and there was no detriment in health-related quality of life [22]. In a post-hoc analysis for the SOLO-1 trial, the median PFS was 56 months (95% CI: 41.9 months not reached) with olaparib versus 14 months with placebo (HR: 0.33; 95% CI: 0.25–0.43) at a median follow-up of five years [23]. Based on the objective evidence from SOLO-1, the FDA approved the olaparib monotherapy as the first-line maintenance treatment for patients with platinum-sensitive advanced (FIGO stage III–IV), high-grade, *BRCA*-associated ovarian cancers in 2018.

### 4.2. Rucaparib

Rucaparib (CO-338; formerly known as AG-014447 and PF-01367338) is an orally available potent small-molecule PARP inhibitor. It was first approved by the FDA in 2016 and by the EMA in 2018 as a single-agent treatment for patients with advanced ovarian cancers associated with deleterious germline or somatic *BRCA* mutations who have received two or more chemotherapies. The efficacy of rucaparib as a monotherapy for the treatment of ovarian cancer was initially assessed in two single-arm trials: the phase I–II trial Study 10 and the phase II trial Study 2 (ARIEL 2).

Study 10, published in 2017, was a three-part, open-label, phase I–II study testing the oral single-agent rucaparib: phase I (part 1) was designed to determine the maximum tolerated dose (MTD), recommended phase II dose (RP2D), and to explore the preliminary efficacy of oral rucaparib administered in 21-day continuous cycles in patients with advanced solid tumors. A total of 56 patients received oral rucaparib (at doses ranging from 40 to 500 mg QD and 240 to 840 mg BID). No MTD was identified using the protocol-specified criteria, and a dose of 600 mg BID was selected as the RP2D based on manageable toxicity and clinical activity. Phase II (part 2A) evaluated the RP2D of oral rucaparib (600 mg BID) in 42 patients with platinum-sensitive, high-grade serous or endometrioid, fallopian tube, or primary peritoneal cancers associated with a germline *BRCA1/2* mutation (gBRCAm). The objective response rate (ORR) was 59.5%, with a median duration of response of 7.8 months (95% CI: 5.6 months–10.5 months). In part 2B, rucaparib was tested in patients with relapsed high-grade ovarian cancer (HGOC) with a germline or somatic *BRCA1/2* mutation (g/sBRCAm) who had received at least three cycles of prior chemotherapy. Part 3 is ongoing and currently assessing the pharmacokinetics and safety profile of a higher dose of rucaparib in patients with a relapsed solid tumor associated with g/s BRCAm [24].

The ARIEL 2 study was a multicenter, two-part, phase II open-label trial investigating the role of rucaparib in recurrent, platinum-sensitive, high-grade ovarian carcinomas after one or more chemotherapy (part 1) and three or four cycles of prior chemotherapy (part 2). The enrollment into ARIEL 2 part 1 is complete, while an extension (part 2) is ongoing. The ARIEL 2 part 1 enrolled 192 patients and classified them into three predefined HRD subgroups: BRCA1/2 mutant (*n* = 40), BRCA wild-type with loss of heterozygosity (LOH) high (LOH high group, *n* = 82), and BRCA wild-type and LOH low (LOH low group, *n* = 70). All patients began to undergo treatment with oral rucaparib at a dose of 600 mg BID for continuous 28-day cycles until disease progression or any other reason for discontinuation. The median PFS after rucaparib treatment was significantly longer in the BRCA-mutated subgroup (12.8 months; HR: 0.27, 95% CI: 0.14–0.44, *p* < 0.0001), and in the LOH high group (5.7 vs. 5.2 months; HR: 0.62, 95% CI: 0.42–0.90, *p* = 0.011) compared with the LOH low group [25].

Further data to support the usage of rucaparib in multiply relapsed diseases come from a pooled analysis of two phase II studies that were undertaken to characterize the antitumor activity and safety profile of the rucaparib in the eligible patients from Study 10 and ARIEL2 who received a starting dose of oral rucaparib 600 mg BID. The analysis focused on the patients with HGOC and a deleterious germline or somatic *BRCA1/2* mutation who received at least two prior chemotherapies: 42 patients were from Study 10 (Part 2A) and 64 from ARIEL 2 (Part 1 and 2). In the efficacy population (*n* = 106), the overall response rate (ORR) was 53.8% (95% CI: 43.8%–63.5%); 8.5% and 45.3% of patients achieved complete and partial responses, respectively. The median duration of response rate (DOR) was 9.2 months (95% CI: 6.6 months–11.6 months). In addition, 74.5% of the patients exhibited sensitivity to their last platinum-based therapy, 18.8% were platinum-resistant, and 8.4% were platinum-refractory. This integrated safety analysis confirmed the manageable toxicity profile of rucaparib [26].

In 2018, the FDA expanded rucaparib indications to the maintenance therapy of recurrent high-grade serous or endometrioid ovarian cancers, fallopian tube cancers, and primary peritoneal cancers, regardless of the presence or absence of BRCA mutations, based on the double-blind, placebo-controlled, phase III ARIEL 3 trial, in which 564 eligible patients had undergone at least two previous platinum-based chemotherapy regimens and achieved complete or partial response to their last platinum-based regimens and were previously PARP-inhibitor-untreated. They were randomly assigned to receive the maintenance therapy with rucaparib (*n* = 375) at a dose of 600 mg BID or placebo (*n* = 189) in a 28-day cycle. In contrast to other studies of PARP inhibitors, patients were permitted to be enrolled in this study even if they had residual bulky diseases (≥2 cm). Across all primary analysis groups, rucaparib significantly improved the PFS in patients with platinum-sensitive ovarian cancers who had achieved a response to platinum-based chemotherapy. Rucaparib significantly improved the PFS among those with a known genomic or somatic *BRCA* mutation (16.6 months vs. 5.4 months; HR: 0.23, 95% CI: 0.16–0.34, *p* = 0.0001). In patients with a HRD subgroup (236 [63%] vs. 118 [62%]), it was 13.6 months vs. 5.4 months (HR: 0.32, 95% CI: 0.24–0.42; *p* < 0.0001). In the intention-to-treat (ITT) population, it was 10.8 months vs. 5.4 months (HR: 0.36; 95% CI: 0.30–0.45; *p* < 0.0001) [27].

Rucaparib has shown PFS benefits when combined with chemotherapy in multiple relapsed diseases, as discussed previously in Study 10 and ARIEL. The latest open-label, randomized, controlled, phase 3 study that was published in 2022, ARIEL 4, supports the usage of rucaparib as an alternative treatment option to chemotherapy for patients with relapsed, BRCA1/2 mutated ovarian cancers who have received at least two cycles of previous chemotherapy regimens. The eligible patients were randomly assigned (2:1) to receive oral rucaparib (600 mg BID) or chemotherapy. Patients assigned to the chemotherapy group with platinum-resistant or partially platinum-sensitive disease were given weekly paclitaxel (starting dose 60–80 mg/m^2^ on days 1, 8, and 15), and those with fully platinum-sensitive disease received platinum-based chemotherapy (single-agent cisplatin or carboplatin, or platinum-doublet chemotherapy). Overall, rucaparib improved the median PFS in comparison to chemotherapy (7.4 vs. 5.7 months, HR: 0.67, 95% CI: 0.52–0.86). Among the patients with platinum-sensitive diseases (progression ≥ 12 months), the PFS was 12.9 months versus 9.6 months for the rucaparib group vs. chemotherapy group (HR: 0.69, 95% CI: 0.37–1.29). Those with partially platinum-sensitive disease (progression during 6–12 months) experienced a PFS of 8.0 months versus 5.5 months (HR: 0.40, 95% CI: 0.24–0.65), respectively. Among those with platinum-resistant disease, the median PFS was 6.4 months versus 5.7 months for rucaparib versus chemotherapy (HR: 0.78, 95% CI: 0.54–1.13) [28]. The results of ARIEL 4 revealed the PFS benefit of rucaparib over chemotherapy when used in relapsed ovarian cancers.

### 4.3. Niraparib

Niraparib (Zejula, TESARO Inc., Waltham, Massachusetts, USA, formerly known as MK-4827) is an orally potent PARP inhibitor and the third FDA-approved PARPi in treating ovarian cancers. In 2017, the FDA approved niraparib for maintenance treatment of all patients with platinum-sensitive, recurrent advanced epithelial ovarian, fallopian tube, and primary peritoneal cancers in complete or partial response to their last platinum-based chemotherapy, irrespective of the BRCA or HRD status based on the randomized, double-blind, phase III NOVA study. A total of 553 eligible patients with recurrent, platinum-sensitive ovarian cancers, fallopian tube cancers, or primary peritoneal cancers with predominantly high-grade serous histologic features were categorized into two independent cohorts on the basis of the presence or absence of a germline BRCA mutation (203 gBRCAm patients and 350 without gBRCAm patients). The patients were randomized to receive niraparib maintenance therapy (300 mg QD) or a placebo after completion of platinum-based chemotherapy until disease progression or unacceptable toxicity. Compared with placebo, niraparib increased the PFS significantly in the experimental niraparib cohorts. Comparing niraparib with placebo, the PFS was 21.0 months vs. 5.5 months in the gBRCAm cohort (HR: 0.27; 95% CI: 0.17–0.41, *p* < 0.001) and 9.3 months vs. 3.9 months in the overall non-gBRCAm cohort. The PFS of the HRD-positive subgroup in the non-gBRCAm patients was 12.9 months vs. 3.8 months (HR: 0.38; 95% CI: 0.24–0.59, *p* < 0.001), while the PFS of the HRD-negative and non-gBRCA mutation subgroup was 6.9 vs. 3.8 months (HR: 0.58; 95% CI: 0.36–0.92; *p* = 0.02) [29].

A multicenter, open-label, single-arm, phase II study, QUADRA, evaluated the safety and activity of the niraparib monotherapy. A total of 463 patients with metastatic, relapsed, high-grade serous (grade 2 or 3) epithelial ovarian, fallopian tube, or primary peritoneal cancers, who had been previously treated with chemotherapy, received oral niraparib 300 mg QD continuously, beginning on day 1 and every cycle (28 days) thereafter until disease progression. In the overall ovarian cancer population, 222 (48%) of 463 patients had HRD-positive tumors (including germlineBRCA-mutated, somatic BRCA-mutated, and non-BRCA-mutated HRD-positive tumors) and 87 (19%) of 463 patients had a germline or somatic BRCA mutation. The median follow-up for overall survival was 12.2 months. In HRD-positive, platinum-sensitive patients who had received ≥3 chemotherapy regimens without prior PARPi therapy, niraparib achieved an ORR of 27.5% (95% CI: 15.9–41.7%), a disease control rate (DCR) of 68.6%, and a DOR of 9.2 months [30].

Based on the results, the FDA has approved the usage of niraparib for patients with relapsed, advanced ovarian, fallopian tube, or primary peritoneal cancers treated with three or more prior chemotherapy whose cancers are associated with HRD-positive status in October 2019.

Finally, niraparib has obtained FDA approval as a maintenance therapy in the first-line setting, irrespective of the tumor BRCA status in 2020. Similar to the SOLO-1 results for the olaparib monotherapy, the randomized, double-blind, phase 3 PRIMA trial has demonstrated a remarkable improvement in the PFS with using the niraparib monotherapy as the maintenance treatment of adult patients with primary advanced epithelial ovarian, fallopian tube, or primary peritoneal cancers who are in complete or partial response to first-line platinum-based chemotherapy. Unlike SOLO-1, PRIMA included patients who did not have deleterious mutations in *BRCA1/2*, and the results showed a significant PFS improvement with the niraparib monotherapy for the overall population, regardless of the presence or absence of HRD [31].

A total of 733 patients with newly diagnosed advanced ovarian cancers were randomly assigned in a 2:1 ratio to receive either niraparib or placebo once daily after a response to platinum-based chemotherapy. A statistically significant improvement in the PFS was noted in the patients randomized to niraparib usage compared with placebo usage in the HRD and overall population. In the HRD population, the median PFS was 21.9 months in the patients receiving niraparib and 10.4 months in those receiving placebo (HR: 0.43; 95% CI: 0.31–0.59; *p* < 0.0001). The median PFS in the overall population was 13.8 months in the patients receiving niraparib and 8.2 months in those receiving placebo (HR: 0.62; 95% CI: 0.50–0.76; *p* < 0.0001). During the 24-month interim analysis, the rate of overall survival was 84% in the niraparib group and 77% in the placebo group (HR: 0.70; 95% CI: 0.44–1.11) [31]. The results of PRIMA revealed the benefit of niraparib usage on the PFS in patients with newly diagnosed advanced ovarian cancers, irrespective of the HRD status.

**Table 2:** A summary of randomized trials of PAPR inhibitors, including olaparib, rucaparib, and niraparib.

PAPR Inhibitors	Study Design: RCTs Phase/Author	Patient Population	Patient Number	Intervention	Results/Outcomes
Olaparib	**Study 42** Phase II/Kaufman, B. (2015) [17]	(1) Platinum-sensitive, advanced ovarian cancer (2) Received at least 3 prior lines of chemotherapy (3) Of whom 193 patients had recurrent ovarian cancer (4) BRCA 1/2 mutated	298	Olaparib 400 mg BID	**(1)Objective response rate (ORR):** 34% (46/137; 95% CI: 0.26–0.46) **(2) Median duration of response (DOR):** 7.9 months (95% CI: 5.6 months–9.6 months)
	**Study 19** Phase II/Ledermann, J. (2014) [19]	(1) Platinum-sensitive, relapsed, high-grade ovarian cancer (2) Received two or more platinum-based regimens and had a partial or complete response to their most recent platinum-based regimen. (3) Independent BRCA 1/2 mutational status	265 (1:1)	Maintenance Olaparib capsule (400 mg BID) vs. Placebo	**(1)Median PFS (months):** 8.4 vs. 4.8 (HR: 0.35; 95% CI: 0.25–0.49; *p* < 0.001) **(2)** **Median OS (months):** 29.7 vs. 29.9 (HR 0.94; 95% CI: 0.63–1.39; *p* = 0.75)
	**SOLO-2** Phase III/Pujade-Lauraine, E. (2017) [20]	(1) Platinum-sensitive, relapsed, high-grade ovarian cancer (2) Received two or more platinum-based regimens and had had a partial or complete response to their most recent platinum-based regimen (3) BRCA 1/2 mutated	295 (2:1)	Maintenance Olaparib tablets (300 mg BID) vs. Placebo	**(1)Median PFS (months):** 19.1 vs. 5.5 (HR: 0.30; 95% CI: 0.22–0.41; *p* < 0.0001) **(2)** **Median OS (months):** 51.7 vs. 38.8 (HR: 0.74; 95% CI: 0.54–1.00; *p* = 0.0537)
	**SOLO-1** Phase III/Moore, K. (2018) [22]	(1) Newly diagnosed, advanced (FIGO stage III–IV), high-grade ovarian cancer (2) Partial or complete clinical response to platinum-based chemotherapy. (3) Mutation in *BRCA1*, *BRCA2*, or both (*BRCA1/2*)	391 (2:1)	Maintenance Olaparib tablets (300 mg BID) vs. Placebo	**(1)** Lower three-year rate of disease progression or death compared with the placebo therapy (60% vs. 27%; HR: 0.30, 95% CI: 0.23–0.41, *p* < 0.0001) **(2)** **Median PFS (months):** 56 (95% CI: 41.9 months not reached) vs. 14 (HR: 0.33; 95% CI: 0.25–0.43)
Rucaparib	**Study 10** Phase I–II/Kristeleit, R. (2017) [24]	(1) Part 1 (phase I) sought to determine the MTD, recommended phase II dose (RP2D), and pharmacokinetics of oral rucaparib administered in 21-day continuous cycles in patients with advanced solid tumors. (2) Part 2A (phase II): platinum-sensitive, high-grade serous or ovarian cancer associated with germline BRCA1/2 mutation and received between 2 and 4 prior treatment regimens (3) Part 2B of this study is currently assessing the efficacy of rucaparib in patients with relapsed HGOC associated with a germline or somatic *BRCA 1/2* mutation who had received at least three prior chemotherapy regimens. (4) Part 3 is ongoing and currently assessing the pharmacokinetic (including the effect of food) and safety profile of a higher dose tablet of rucaparib in patients with a relapsed solid tumor associated with a germline or somatic BRCA1/2 mutation.	Part 1: 56 Part 2: 42	Part 1: Rucaparib 40~500 mg QD and 240~840 mg BID Part 2A: Rucaparib 600 mg BID	**(1)** Part 1: No maximum tolerated dose (MTD) was identified using the protocol- specified criteria, and a dose of 600 mg BID was selected as the RP2D based on manageable toxicity and clinical activity **(2)** Part 2: **Objective response rate (ORR):** 59.5% with a median duration of response of 7.8 months (95% CI: 5.6–10.5 months)
	**ARIEL 2** Phase II/Swisher, E. M. (2017) [25]	(1) Recurrent, platinum-sensitive, high-grade ovarian carcinomas after one or more chemotherapy (part 1) and 3 or 4 cycles of prior chemotherapy (part 2) (2) BRCA 1/2 mutated	Part 1: 192	Rucaparib at a dose of 600 mg BID	**(1)Median PFS:** BRCA-mutated subgroup (12.8 months; HR: 0.27, 95% CI: 0.14–0.44, *p* < 0.0001) **(2)** LOH high group (5.7 vs. 5.2 months; HR: 0.62, 95% CI: 0.42–0.90, *p* = 0.011) compared with the LOH low group
	**ARIEL 3** Phase III/Coleman, R. L. (2017) [27]	(1) Recurrent, platinum-sensitive, high-grade serous or endometrioid ovarian cancer (2) Received at least two previous platinum-based chemotherapy regimens, had achieved complete or partial response to their last platinum-based regimen (3) Independent BRCA 1/2 mutational status	564 (2:1)	Maintenance Rucaparib tablets 600 mg BID vs. placebo	**Median PFS (months):** **Overall population:** 10.8 vs. 5.4 (HR: 0.37; 95% CI: 0.30–0.45; *p* < 0.0001) **BRCAm population:** 16.6 vs. 5.4 (HR: 0.23, 95% CI: 0.16–0.34, *p* = 0.0001) **HRD (+) population:** 13.6 vs. 5.4 (HR: 0.32, 95% CI: 0.24–0.42; *p* < 0.0001) **Intention-to-treat population:** 10.8 vs. 5.4 (HR: 0.36; 95% CI: 0.30–0.45; *p* < 0.0001)
	**Ariel 4** Phase III/Kristeleit, R. (2022) [28]	(1) Relapsed, ovarian cancers who have received at least two cycles of previous chemotherapy regimens. (2) BRCA1/2 mutated	394 (2:1)	Rucaparib 600 mg BID vs. chemotherapy (weekly paclitaxel or platinum-based)	**Median PFS (months):** **Overall population:** 7.4 vs. 5.7 (HR: 0.64, 95% CI: 0.49–0.84; *p* = 0.0010) **Intention-to-treat population:** 7.4 vs. 5.7 (HR: 0.67; 95% CI: 0.52–0.86; *p* < 0.0017)
Niraparib	**NOVA study** Phase III/Mirza, M.R (2016) [29]	(1) Recurrent, platinum-sensitive ovarian cancers with predominantly high-grade serous histologic features. (2) gBRCA cohort and non-gBRCA cohort	553 (2:)	Maintenance Niraparib 300 mg QD vs. Placebo	**Median PFS (months):** **gBRCAm cohort:** 21.0 vs. 5.5 (HR: 0.27; 95% CI: 0.17–0.41; *p* < 0.001) **HRD-positive and non-gBRCAm:** 12.9 vs. 3.8 (HR: 0.38; 95% CI: 0.24–0.59; *p* < 0.001) **HRD-negative and non-gBRCAm:** 6.9 vs. 3.8 (HR: 0.58; 95% CI: 0.36–0.92; *p* = 0.02) **Median OS (months):** **gBRCAm cohort:** 45.9 vs. 43.2 (restricted mean) **non-gBRCAm cohort:** 38.5 vs. 39.1(restricted mean)
	QUADRA Phase II/Moore, K. N. (2019) [30]	(1) Metastatic, relapsed, high-grade serous epithelial ovarian cancer (2) Received treatment with three or more previous chemotherapy regimens. (3) Homologous recombination deficiency (HRD)-positive tumors (including patients with/without *BRCA* mutations)	463	Niraparib 300 mg QD continuously, beginning on day 1 and every cycle (28 days) thereafter until disease progression.	**Objective response rate (ORR):** 27.5% (95% CI: 15.9%–41.7%) **Disease control rate (DCR):** 68.6% **Duration of response (DOR):** 9.2 months
	**PRIMA study** Phase III/González-Martín, A. (2019) [31]	(1) Newly diagnosed advanced ovarian cancers (2) Complete or partial response to platinum-based chemotherapy (3) Independent BRCA 1/2 mutational status	733 (2:1)	Maintenance Niraparib tablets (300 mg BID) vs. Placebo	**Median PFS (months):** **Overall population:** 13.8 vs. 8.2 (HR: 0.62; 95% CI: 0.50–0.76; *p* < 0.0001) **HRD (+) population:** 21.9 vs. 10.4 (HR: 0.43; 95% CI: 0.31–0.59; *p* < 0.0001) **HRD (+), BRCAm population:** 22.1 vs. 10.9 (HR: 0.40; 95% CI: 0.27–0.62) **HRD (+), non-BRCAm population:** 19.6 vs. 8.2 (HR: 0.50; 95% CI: 0.31–0.83) **HRD (-) population:** 8.1 vs. 5.4 (HR: 0.68; 95% CI: 0.49–0.94) **HRD-unknown population:** median not reported (HR: 0.59; 95% CI: 0.51–1.43)

## 5. PARP Inhibitors and Antiangiogenic Agents

Angiogenesis is the process of new blood vessel formation that plays an important role in both normal ovarian physiology and ovarian cancer progression [32]. Vascular endothelial growth factors (VEGF)s A–D and their receptors (VEGFR)s 1–3 regulate angiogenesis, which is expressed at varying levels on EOC cells. Increased VEGF signaling has been associated with the development of malignant ascites and tumor progression [32,33]. Antiangiogenic therapies induce a hypoxic cellular state leading to the downregulation of HR repair genes (BRCA1, BRCA2, and RAD51) [34]. Angiogenesis inhibitors, such as bevacizumab and cediranib, have demonstrated antitumor activity in ovarian cancers. The monoclonal antibody bevacizumab targets VEGF-A and the small-molecule inhibitor cediranib targets multiple factors, including VEGFRs 1–3 and c-Kit [35]. Bevacizumab, in combination with platinum-based chemotherapy, followed by bevacizumab alone as maintenance, has been approved by the FDA for treatment of patients with advanced EOCs after initial surgical intervention, following the results of the GOG-0218 [36] and ICON-7 studies [37].

The FDA approval for olaparib plus bevacizumab as maintenance therapy for patients with newly diagnosed, advanced, high-grade ovarian cancers who have responded to first-line platinum-based chemotherapy plus bevacizumab is supported by the results of the PAOLA-1 trial [38]. In this randomized, double-blind, international phase 3 trial, patients were randomly assigned to two groups of maintenance bevacizumab (15 mg/kg Q21D) with or without olaparib (300 mg BID). The trial reported that in the patients with advanced ovarian cancers who had received first-line standard therapy, including bevacizumab, the addition of maintenance olaparib provided a significant PFS benefit. The benefit was substantial in the patients with HRD-positive tumors, including those without BRCA mutations. After a median follow-up of 22.9 months, a statistically significant improvement was observed in the median PFS for the patients who received olaparib plus bevacizumab versus bevacizumab alone plus placebo (22.1 months vs. 16.6 months; HR: 0.59; 95% CI: 0.49–0.72; *p* < 0.001). The HRs (Olaparib Group vs. Placebo Group) for disease progression or death were 0.33 (95% CI: 0.25–0.45) in the patients with HRD-positive tumors that had BRCA mutations (the median PFS: 37.2 months vs. 17.7 months), and 0.43 (95% CI: 0.28–0.66) in the patients with HRD-positive tumors that did not have BRCA mutations (the median PFS: 28.1 months vs. 16.6 months), respectively. Among the subgroups with BRCA-positive ovarian cancers, the combination of olaparib plus bevacizumab improved the PFS than bevacizumab alone (37 months vs. 22 months; HR: 0.31, 95% CI: 0.20–0.47) [38]. As maintenance therapy, the combination of bevacizumab and olaparib appears beneficial for patients with advanced, high-grade ovarian cancers. However, their synergistic effects remain to be investigated due to the relatively small sample size of the existent studies.

## 6. Results and Discussion

EOC remains the most lethal gynecologic malignancy in the United States. The mainstay for the treatment of newly diagnosed EOC is surgical debulking and combined taxane- and platinum-based chemotherapy. For patients who have stage II–IV diseases at presentation, options following surgery and chemotherapy depend on the success of these interventions. These patients should be evaluated with imaging as clinically indicated to determine the extent of residual disease or progression and screen for new metastases. For those who are in complete clinical remission, partial remission, or stable disease, recommended options also depend on the extent of their response and the types of primary chemotherapies they received. All patients with the diagnoses of ovarian, fallopian tube, or peritoneal cancers should have a genetic risk evaluation, irrespective of their family history, as the results may impact treatment decisions too. A maintenance therapy with PARP inhibitors can be considered for chemo-sensitive, symptom-free patients if the results of genetic tests are confirmed. Usually, the responses to platinum-based chemotherapy are high, but the vast majority of women will experience a relapse of their cancer following a median PFS of approximately 12 months and require more treatment. In patients with advanced and recurrent ovarian cancers, targeted therapies, including antiangiogenic treatment and PARP inhibitors, have been investigated with the aim of prolonging the PFS and OS.

PARP inhibitors work based on the concept of synthetic lethality. For ovarian cancers associated with *BRCA* mutations or HRD, the targeted therapy result in cell death due to inefficiencies in DNA cell repair mechanisms. As we have mentioned above, several PARP inhibitors have been shown to be active in recurrent ovarian cancers and have been FDA approved for multiple indications in ovarian cancers. To date, no direct trial comparing the three FDA-approved commercially available PARP inhibitors has been performed. The selection of the PARP inhibitors remains a challenge and depends on personal preference. At the most, the advantage of niraparib usage lies in the benefit for the PFS and OS in the patients with newly diagnosed advanced ovarian cancers, irrespective of the HRD status. Grossly, PARP inhibitors have demonstrated some therapeutic benefits in many randomized phase II and III trials when combined with the standard CRS for advanced EOCs. However, there are still some limitations to the usage of PARP inhibitors in clinical practice. Among them, drug resistance is the major obstacle to PARP inhibitors, and further surveys for drug resistance and toxicity are suggested.

In the current setting, no consensus exists on the protocol to be used. This includes variation in the choice of PARP inhibitors, dosage, length of time, and timing of surgery. Similarly, different results exist in the past studies that investigated the usage of PARP inhibitors for primary or recurrent advanced ovarian cancers. In practice, several discrepancies in results and conclusions among these studies may originate from the clinical setting: the criteria, dosage, and duration of PARP inhibitors.

In general, all currently available PARP inhibitors have some common adverse events (AEs) that fall into the categories of hematologic, gastrointestinal, respiratory, cardiovascular events, and others, which can compromise the quality of life of patients. In particular, the most common non-hematologic AEs include nausea, vomiting, diarrhea, constipation, and fatigue, while hematologic AEs include anemia, thrombocytopenia, neutropenia, and myelosuppression. Across the phase III trials—the SOLO-1 trial (olaparib monotherapy 300 mg BID vs. placebo), PAOLA-1 trial (olaparib 300 mg BID plus bevacizumab vs. bevacizumab monotherapy), and PRIMA trial (niraparib monotherapy 300 mg QD vs. placebo)—all PARP inhibitors as an FDA-approved maintenance therapy following the first-line platinum-based therapy have shown higher rates of a number of common non-hematologic AEs, such as nausea, vomiting and fatigue/asthenia that are generally in low-grade and rarely lead to study-drug discontinuation. Similar to bevacizumab, niraparib is associated with a risk of hypertension (>grade 3). Anemia and neutropenia are the most common high-grade hematologic AEs (grade ≥ 3) for single-agent PARP inhibitor maintenance therapy compared with placebo and are the most common cause of dose discontinuation. Although rare (≤2%), PARP inhibitor therapy is also associated with a risk of myelodysplastic syndrome or acute myeloid leukemia. Across all trials, PARP inhibitors are well tolerated, and there are no statistically significant differences between treatment arms in the health-related QOL metrics evaluated [22,31,38].

Thrombocytopenia is a peculiar hematologic AE in the treatment of niraparib, which has been reported in both the Nova trial and PRIMA trial (both niraparib dosing at 300 mg QD), and the most frequent treatment-related emergency event that requires dose reduction. Two factors have been identified as potential predictors of myelosuppression: baseline platelet count and baseline body weight. Patients who have a baseline platelet count < 150,000/μL and/or body weight < 77 kg when niraparib is started are at a higher risk of grade 3 or higher thrombocytopenia and are likely to need subsequent dose reduction. It is recommended that patients who meet either of these criteria should start dosing at 200 mg/daily. Dose escalation may be considered with close monitoring of blood counts if no hematologic events occur within the first 2–3 months of dosing. The PFS in the patients who are dose-reduced to either 200 or 100 mg is consistent with that of the patients who remain at the 300 mg starting dose. Niraparib also causes hypertension, tachycardia, and headaches due to its inhibition of dopamine, norepinephrine, and serotonin transporters. The patients affected by hypertension have to monitor their blood pressure regularly and should keep blood pressure maintained below 120/80 mmHg [39,40].

Overall, it seems that the PARP inhibitors can extend the life span of patients with epithelial ovarian cancers but cannot eradicate the cancers that have developed after treatment with cisplatin/paclitaxel. Complete surgical removal (cytoreduction) of residual ovarian tumors from the peritoneal cavity seems to be the only successful way to eliminate the tumors, but this is rarely successful due to the difficulty in doing the cytoreductive surgery in the peritoneal space. Moreover, ovarian cancer cells, similar to many other types of cancer cells, have devious mechanisms to evade stress, which is mediated by drugs or hypoxia. In high-grade serous ovarian cancers (HGSOC), the cancer cells under stress have the capacity to escape from stress and to become polyploidy giant cancer cells (PGCCs), which eventually release daughter cells that are drug-resistant [41]. As stem-like, self-renewing cells, the PGCCs can survive the attack of chemotherapeutic agents and start endoreplication, in which process these cells have mutations and subsequently altered expression/function of kinases, p53, and stem cell markers. The generated diploid daughter cells can exhibit drug resistance and facilitate tumor growth and metastasis [41]. Thus, developing drugs to target PGCCs and endoreplication may be an important approach to reducing the appearance of drug-resistant progeny.

It remains not fully understood with regard to the molecular and cellular mechanisms of EOCs and their targeted therapies. Further efforts are still required to investigate the therapeutic role of PARP inhibitors in ovarian cancers. To minimize the heterogeneity of research in the future, the standardization of several important factors in EOCs and relevant treatment should be considered. Critical factors include the timing, dosage, and duration of PARP inhibitors, all of which have significant impacts on the therapeutic effects. Furthermore, the severity and outcomes in the patients with advanced EOCs also need standardization. Moreover, a larger sample size is required to obtain a reliable conclusion and to improve the reproducibility of the research results.

## 7. Conclusions

The treatment of ovarian cancer remains a challenge, and the majority of patients with advanced-stage ovarian cancers experience relapse and require additional treatment despite initial therapy, including an optimal cytoreductive debulking surgery and platinum-based chemotherapy. Targeted therapy at DNA repair genes has become a unique strategy to combat HRD cancers in recent years. Poly (ADP-ribose) polymerase (PARP), a family of proteins, plays an important role in DNA damage repair, genome stability, and apoptosis of cancer cells, especially in HRD cancers. PARP inhibitors have been reported to be highly effective and low-toxicity drugs that benefit patients with HRD (i.e., BRCA 1/2 mutated) ovarian cancers by blocking the DNA repair pathways and inducing apoptosis of cancer cells.

PARP inhibitors compete with NAD^+^ at the catalytic domain (CAT) of PARP to block PARP catalytic activity and the formation of PAR polymers. These effects compromise the cellular ability to overcome DNA SSB damage. On the other hand, the unrepaired SSBs can be converted to DSBs through the collapse of the replication fork when PARP is inhibited (PARP trapping mechanism). Homologous recombination (HR) is the essential error-free pathway to repair DNA DSBs during cell replication, and it requires functional BRCA 1/2 proteins. The process of HR will be blocked in the condition of BRCA 1/2 mutations. The PARP-associated HR pathway can also be partially interrupted by using PARP inhibitors.

At present, three PARP inhibitors, including olaparib, rucaparib, and niraparib, have been approved by the FDA for clinical use in EOC patients, though with different clinical indications and toxicity profiles. Grossly, PARP inhibitors have demonstrated some therapeutic benefits in many randomized phase II and III trials when combined with the standard CRS for advanced EOCs. This review could help the researchers and healthcare practitioners understand the recent evidence regarding the usage of PARP inhibitors for advanced EOCs, suggestive of future developments in this emerging area. Nevertheless, many unknown aspects of PARP inhibitors, including detailed mechanisms of action, along with the effectiveness and safety for the treatment of epithelial ovarian cancers, warrant further investigation.

## Figures and Tables

**Table 1:** The presence or deficiency of HR and BER on the outcomes of normal or BRCA 1/2 mutated cells treated with/without PARP inhibitors.

Conditions	HR	BER	Outcomes
Cancer cells (no BRCA mutation) without treatment	+	+	Cell survival
Cancer cells (no BRCA mutation) treated with PARPi	+	−	Cell survival
BRCA-mutated (HRD) cancer cells without treatment	−	+	Cell survival
BRCA-mutated (HRD) cancer cells treated with PARPi	−	−	Cell death

## Data Availability

Not applicable.
